# Molecular Identification of Non-tuberculous Mycobacteria in Suspected Tuberculosis Cases in Central India

**DOI:** 10.7759/cureus.39992

**Published:** 2023-06-05

**Authors:** Malti Dadheech, Anvita Gupta Malhotra, Sakshi Patel, Jitendra Singh, Sagar Khadanga, Alkesh Khurana, Shashank Purwar, Debasis Biswas, Sarman Singh, Anand K Maurya

**Affiliations:** 1 Department of Microbiology, All India Institute of Medical Sciences, Bhopal, Bhopal, IND; 2 Department of Translation Medicine, All India Institute of Medical Sciences, Bhopal, Bhopal, IND; 3 Department of General Medicine, All India Institute of Medical Sciences, Bhopal, Bhopal, IND; 4 Department of Pulmonary and Critical Care Medicine, All India Institute of Medical Sciences, Bhopal, Bhopal, IND; 5 Department of Biological Sciences, Indian Institute of Science Education and Research, Bhopal, Bhopal, IND

**Keywords:** mycobacterium abscessus, line probe assay, molecular diagnosis, mycobacterium fortuitum, nontuberculous mycobacteria, mycobacterium tuberculosis

## Abstract

Introduction

*Mycobacterium tuberculosis* complex (MTBC), the primary cause of tuberculosis (TB), must be accurately identified to implement effective patient management and control strategies. Non-tuberculous mycobacteria (NTM) in suspected TB cases can result in erroneous diagnoses and needless treatment.

Objective

The study aimed to identify NTM in patients suspected of TB at a tertiary care hospital in central India using molecular methods.

Methods

This prospective study enrolled 400 suspected pulmonary and extra-pulmonary TB patients. Patients between the age of two to 90 years, of either gender, new and previously treated cases, Culture positive, patients with immune-compromised status, patients not responding to ATT, HIV positive and negative, and willing to give consent were included in the study. Liquid culture via the Mycobacterial growth indicator tube (MGIT) system was used to culture mycobacteria from clinical samples. The SD Bioline Ag MPT64 Test (Standard Diagnostics, South Korea) and in-house multiplex-PCR (mPCR) were used to differentiate between Mycobacterium tuberculosis complex and NTM species for the molecular identification of NTM GenoType® Mycobacterium Common Mycobacteria (CM) assay kit (HAIN Life Science, Nehren, Germany) was used following the manufacturer's protocol.

Results

Only 59/400 (14.7%) of the samples produced a positive result in MGIT culture, indicating the presence of mycobacteria, and 85.25% of the remaining 341 samples were negative for mycobacterial growth. Further investigation of these 59 cultures with mPCR and SD Bioline Ag MPT64 test showed that 12 (20.33%) cultures were determined to be NTM, while the remaining 47 (79.67%) were identified as MTBC. Genotype characterization with GenoType® mycobacterium CM assay kit revealed that five of the 12 NTM isolates (41.67%) showed patterns that were consistent with *Mycobacterium* (*M.) fortuitum*, three (25%) showed patterns that were consistent with *M. abscessus*, and four (33.33%) showed patterns that were consistent with *M. tuberculosis*.

Conclusion

These results emphasize the value of molecular methods for precisely identifying mycobacterial species, particularly in suspected TB cases. The high prevalence of NTM in positive cultures emphasizes the significance of differentiating between MTBC and NTM to prevent misdiagnosis and ensure proper care. Understanding the epidemiology and clinical significance of these organisms in central India is made possible by the identification of particular NTM species.

## Introduction

Nontuberculous mycobacteria (NTM), also called mycobacteria other than tuberculosis or environmental mycobacteria, belong to the genus Mycobacteria [1]. NTM are typically free-living organisms that can be found widely distributed in the environment [2]. NTM infections are a growing concern in both pulmonary and extrapulmonary infections in India and worldwide [2,3]. To date, around 200 species of NTM have been identified, and they are capable of causing a variety of pulmonary and extrapulmonary NTM infections [4]. The most common type of NTM infection is pulmonary, accounting for 65-90% of cases. In recent years, NTM has become increasingly significant as human pathogens, with their incidence and prevalence rising globally [2,5]. Pulmonary NTM infections are becoming more prevalent in India [6]. The most commonly isolated NTM species in pulmonary infections are *Mycobacterium avium* complex (MAC), *Mycobacterium kansasii*, and *Mycobacterium abscessus* [7]. NTM species, such as *Mycobacterium (M.) fortuitum*, *Mycobacterium chelonae*, and *Mycobacterium abscessus* are often implicated in these infections, commonly extrapulmonary NTM infections [2,8]. The risk of NTM infections in Individuals with pre-existing lung conditions, such as bronchiectasis, chronic obstructive pulmonary disease (COPD), and cystic fibrosis, are at an increased risk of developing NTM infections, Immunocompromised individuals due to conditions like HIV/AIDS, cancer, autoimmune diseases, and environmental sources of NTM, such as soil, water, and dust, can also increase the risk of infection. Patients who take immunosuppressive medication, are at an increased risk of NTM infections misdiagnosed as TB, which is higher in these regions, leading to underreporting of NTM prevalence [7,9,10]. The prevalence of NTM varies widely across different geographic areas and populations. In India, the reported prevalence of NTM in pulmonary disease ranges from 4% to 27%, with higher rates reported in the southern and northeastern regions [2,6]. Extrapulmonary NTM infections are less common, but there have been reports of NTM causing infections in various body sites such as skin, lymph nodes, and bone [8]. Globally, NTM infections are increasingly recognized as an important cause of chronic lung disease and other infections. In the United States, the incidence of NTM pulmonary disease has significantly increased [8]. In Europe, the incidence of NTM infections varies widely, with higher rates reported in countries [11,12]. In Asia, the prevalence of NTM in pulmonary disease has been reported to be as high as 45% in some regions [13]. The American Thoracic Society and the Infectious Diseases Society of America (ATS/IDSA) have developed diagnostic guidelines for NTM infection. An accurate clinical and laboratory diagnosis is crucial, as different drug treatments are required for each infection [14]. The diagnostic tools, such as Ziehl-Neelsen (ZN) staining and Gene-Xpert MTB/RIF assay/TrueNat MTB assay, cannot detect NTM and speciate NTM species. Other methods, like biochemical and phenotypic methods, are time-consuming and complicated [2,15]. Although various other molecular techniques are available, they are sophisticated and require expensive equipment [15]. The line probe assay (LPA) is a technology named GenoType® Mycobacterium common mycobacteria (CM) assay by Hain Lifescience in Nehren, Germany, which uses this LPA technology to differentiate and identify various species of NTM from cultures [15,16]. This assay can improve patient outcomes by allowing earlier initiation of appropriate treatment. The study aims to identify NTM in patients suspected of TB at a tertiary care hospital in central India using molecular methods.

## Materials and methods

Study design

The prospective study was conducted in the Department of Microbiology at All India Institute of Medical Sciences (AIIMS), Bhopal, India, from February 2020 to December 2020.

Ethical considerations 

The study was evaluated and approved by the Institutional Human Ethics Committee of All India Institute of Medical Sciences, Bhopal (Ref. No: IHEC/LOP/2019/EF0150). The Institutional Ethics Committee's approval ensures that the study was conducted ethically and that the rights and welfare of the participants were protected. Informed consent was taken from all enrolled patients, and their data were kept confidential.

Clinical specimens and data collection

This prospective study enrolled 400 suspected pulmonary and extra-pulmonary TB patients. Patients between the age of two and 90 years, of either gender, new and previously treated cases, culture-positive, patients with immune-compromised status, patients not responding to antitubercular treatment (ATT), HIV positive and negative, and willing to give consent were included in the study. Two to 10 mL of respiratory samples, urine, and body fluids specimens were collected from pulmonary and extrapulmonary sources of individuals suspected of having TB and presenting to the general medicine, pulmonary medicine, and other outpatient and indoor patient departments, were sent for laboratory diagnosis to the TB and molecular medicine laboratory in the department of microbiology, AIIMS Bhopal, India.

Sputum specimens were collected for pulmonary patients, and in cases where patients could not produce sputum, induced sputum and bronchoalveolar lavage (BAL) fluid specimens were obtained for diagnosis. In cases where patients could produce sputum, two samples were obtained separately for testing. Extrapulmonary specimens, such as pus, lymph node aspirate and biopsy, pleural, pericardial, cerebrospinal fluids, and bone marrow, were obtained and processed accordingly.

Microbiological processing of specimens

Biosafety level-II precautions were taken for the processing of clinical samples. Direct acid-fast bacilli (AFB) smear microscopy was performed on each sample by the ZN staining method using sulfuric acid as a decolorizer, and the smear was evaluated under a light-emitting diode microscope using a 100X oil immersion lens. The remaining sample was processed using the standard decontamination protocol (NALC-NAOH method) and inoculated onto liquid culture Mycobacterial growth indicator tube (MGIT) media [17]. The MGIT 960, an automated isolation system, was used for incubation. The MGIT tube contained 7 ml of 7H9 Middlebrook medium, supplemented with 0.8 ml of oleic acid-albumin-dextrose-catalase (OADC) and polymyxin B-amphotericin B-nalidixic acid-trimethoprim azlocillin (PANTA). It was inoculated with 0.5 ml of the decontaminated sample.

To differentiate between MTBC* *and NTM, an immunochromatographic assay (ICA) kit SD Bioline Ag MPT64 TB test (Standard Diagnostics, South Korea) was used on positive culture isolates. To perform this test, 100 µL of culture broth was added to the well of the SD Bioline Ag MPT64 TB test kit cassette in the Class II biosafety cabinet. Inoculated cassettes were left at room temperature for 20 minutes before being checked for control (C) and test (T) bands. The band in the region "C" confirmed the validity of the test. A band in the "T" region was considered positive for MPT64 Antigen. No band in the "T" region was considered negative for MPT64 antigen. No band in the “C” region was considered an invalid test. The negative culture isolates of the SD Bioline Ag MPT64 test were further subjected to NTM species identification [15].

DNA extraction

The MGIT was positive, and ICT-negative cultures were subjected to DNA extraction by the Qiagen MiniAmp DNA extraction kit (Germany) [18]. The quality and quantity of DNA were analyzed by a spectrophotometer (Thermo Scientific NanoDrop 2000; Thermo Fisher Scientific, Waltham, Massachusetts).

In-house multiplex PCR for the identification of NTM species

The in-house multiplex polymerase chain reaction (m-PCR) was performed on 12 culture isolates that tested negative from the SD Bioline Ag MPT-64 test. This is a conventional PCR method that uses three targets: 1) hsp65 for Mycobacterial genus detection, 2) cfp for MTB complex detection, 3) 16S-23S internal transcribed spacer (ITS) sequences for *M. avium *complex detection [19]. The PCR reaction was prepared using the prime pair, dNTPs, Taq polymerase, mgcl2, buffer, and nuclease-free water. The template DNA was added and the reaction mixture was subjected to the PCR. The PCR was performed using the XP ThermoCycler system (Bioer Technology, Italy). The mPCR was performed on DNA extracted from all the SD Bioline Ag MPT64 test-negative isolates.

Agarose gel electrophoresis

First, 1.8% Agarose gel was prepared. Amplicons and 100bp DNA ladder were loaded in the wells. A running buffer was added to cover the surface of the gel. The power supply was programmed to desired voltage (70-90V), and the gel was allowed to run. Bands were visualized at 196bp region for *cfp10* and at 441bp region for *hsp65* primers, and 144bp for MAC under the UV transilluminator.

Genotyping of the NTM

The LPA was used to identify NTM isolates following the manufacturer's protocol, using the GenoType® Mycobacterium Common Mycobacteria (CM) kit (Hain LifeScience, Germany). The LPA was performed on SD Bioline Ag MPT64 test-negative isolates using reagents supplied with the GenoType® Mycobacterium CM assay kit on a PCR thermocycler (Applied Biosystems Model 2720, Waltham, Massachusetts), and hybridization and detection were done manually using a GT-Blot 48 (Hain Lifescience GmbH). A standard strain of *M. kansasii* was used as a positive control, and molecular-grade water was used as a negative control. The NTM species were interpreted and identified based on the manufacturer's instructions, and all procedures were performed following standard operating procedures (SOP) and manufacturer instructions. For operational reasons, pulmonary TB (PTB) patients were defined according to the previous national guidelines [20], regardless of their TB treatment history (new, relapse, treatment failure, and follow-up). Clinically significant NTM were defined as NTM species identified from PTB patients and meeting the ATS/IDASA NTM diagnosis criteria [14].

## Results

**Figure 1:**
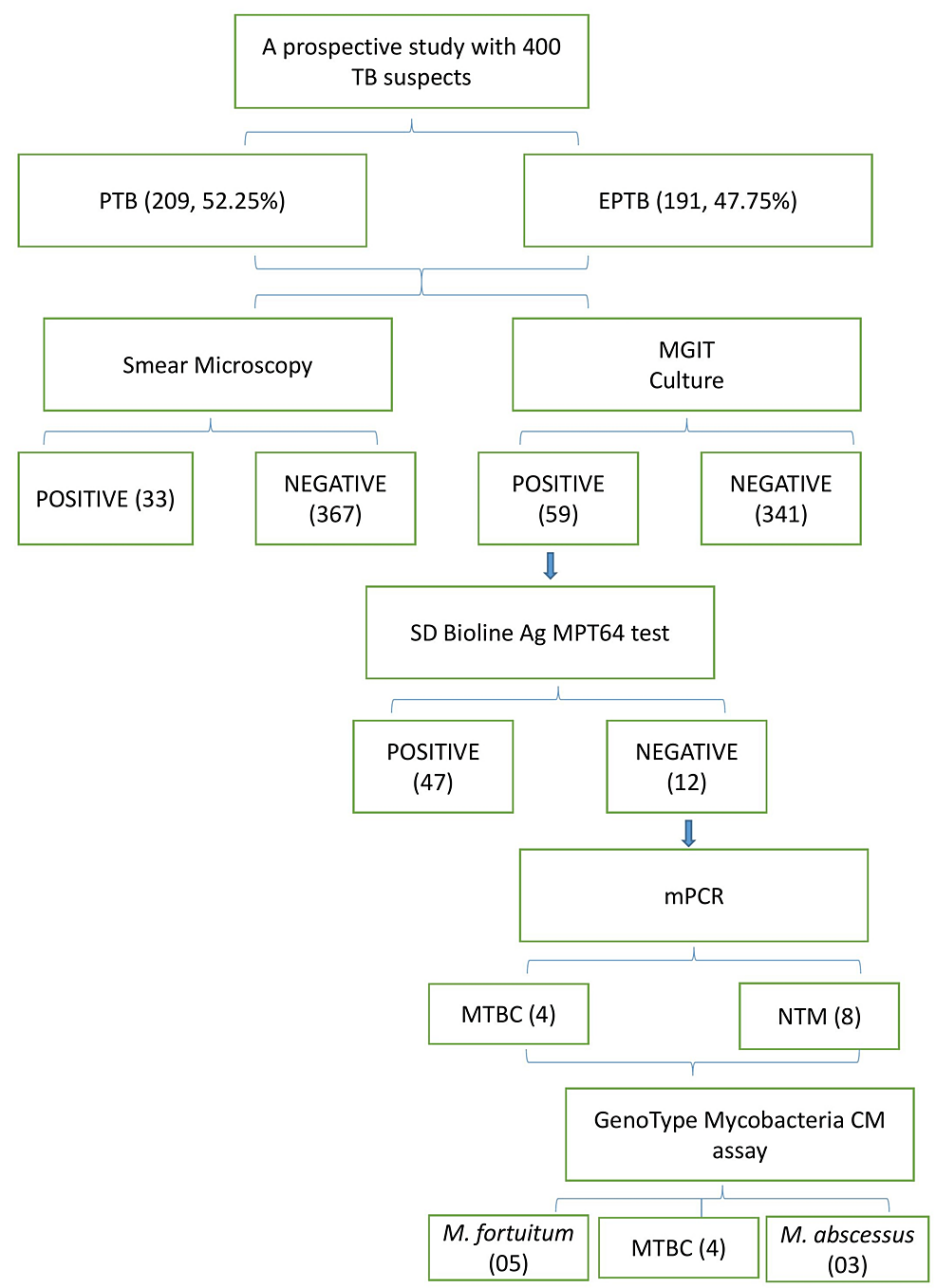
Flow chart of the results

In this study, we identified species of NTM using 400 clinical samples obtained from individuals suspected of having pulmonary and extra-pulmonary TB. Of the 400 samples, 209 (52.25%) were derived from pulmonary samples while the remaining 191 (47.75%) originated from extra-pulmonary sources. The demographic and clinical characteristics of the patients are summarized in Table 1. The flow chart of the results is illustrated in Figure 1.

**Table 1:** The demographic and clinical characteristics of the suspected TB patients

Demographic and clinical parameters		Total (n=400)
Age	0-16 years	94 (23.5%)
	17-32 years	122 (30.5%)
	33-48 years	79 (19.75%)
	49-64 years	75 (18.75%)
	>65 years	30 (7.5%)
Gender	Male	242 (60.5%)
	Female	158 (39.5%)
Signs and Symptoms	Fever	183 (45.75%)
	Cough	97 (24.25%)
	Expectoration	88 (22%)
	Loss of appetite	27 (6.75%)
	Chest pain	45 (11.25%)
	Pleuritic pain	13 (3.25%)
	Weight Loss	35 (8.75%)
	Pleural Effusion	20 (5%)
	CNS infection	30 (7.5%)
	Lymph Node Enlargement	12 (3%)
	Abdominal Pain& Ascites	35 (8.75%)
Previous history of TB	History of TB	09 (2.25%)
	History of ATT	09 (2.25%)
HIV	Negative	400 (100%)
	Positive	0 (0%)
BCG Scar	Present	390 (97.5%)
	Absent	10 (2.5%)
Sample type	Sputum	151 (37.75%)
	Gastric Aspirate	32 (8%)
	CSF	53 (13.25%)
	BAL	06 (1.5%)
	Pleural Fluid	23 (5.75%)
	Lymph Node Aspirate	11 (2.75%)
	Urine	08 (2%)

Among 400 clinical samples, the smear positivity rate determined by microscopy using ZN staining was 33 out of 400 (8.25%). Only 59 out of 400 (14.75%) showed a positive result in the MGIT culture while the remaining 341 out of 400 (85.25%) were negative in the MGIT culture, as indicated in Table 2.

**Table 2:** Results of different diagnostic methods used for the identification of NTM NTM: non-tuberculous mycobacteria

Diagnostic methods (test performed)	Results	Number (%)
Ziehl-Neelsen staining (400)	Positive	33/400 (8.25%)
	Negative	367/400 (91.75%)
MGIT culture (400)	Positive	59/400 (14.75%)
	Negative	341/400 (85.25%)
SD Bioline Ag MPT-64 Test (59)	Positive for MTBC	47/59 (79.67%)
	Negative	12/59 (20.33%)
In-house Multiplex PCR (12)	Positive for cfp10 and hsp65 (MTBC)	04/12 (33.33%)
	Positive for hsp65 (Mycobacterium genus)	08/12 (66.67%)
	Positive for 16S-23S ITS (MAC)	0/12 (0%)
GenoType® Mycobacterium CM assay (12)	MTBC	04/12 (33.33%)
	M. fortuitum	05/12 (41.67%)
	M. abscessus	03/12 (25%)

For rapid differentiation of the MTBC and NTM, the SD Bioline Ag MPT64 test was performed on 59 culture-positive isolates. Of 59 isolates, 47/59 (79.66%) tested positive and confirmed as MTBC and the remaining 12/59 (20.34%) isolates tested negative, which could fall under NTM species as shown in Table 2.

Out of the 12 SD Bioline Ag MPT64 negative isolates, 4/12 (33.33%) were positive for the hsp65 and cfp10 genes (confirmed as MTBC) and the remaining 8/12 (66.67%) were positive for hsp65, found to be NTM. No bands were seen for the MAC gene.

The final confirmation and differentiation of NTM species were carried out using the GenoType® Mycobacterium CM assay, which served as the confirmatory (gold standard) test. This assay was performed on 12 isolates that had tested negative for the SD Bioline Ag MPT64 test, and usable results were obtained.

**Figure 2:**
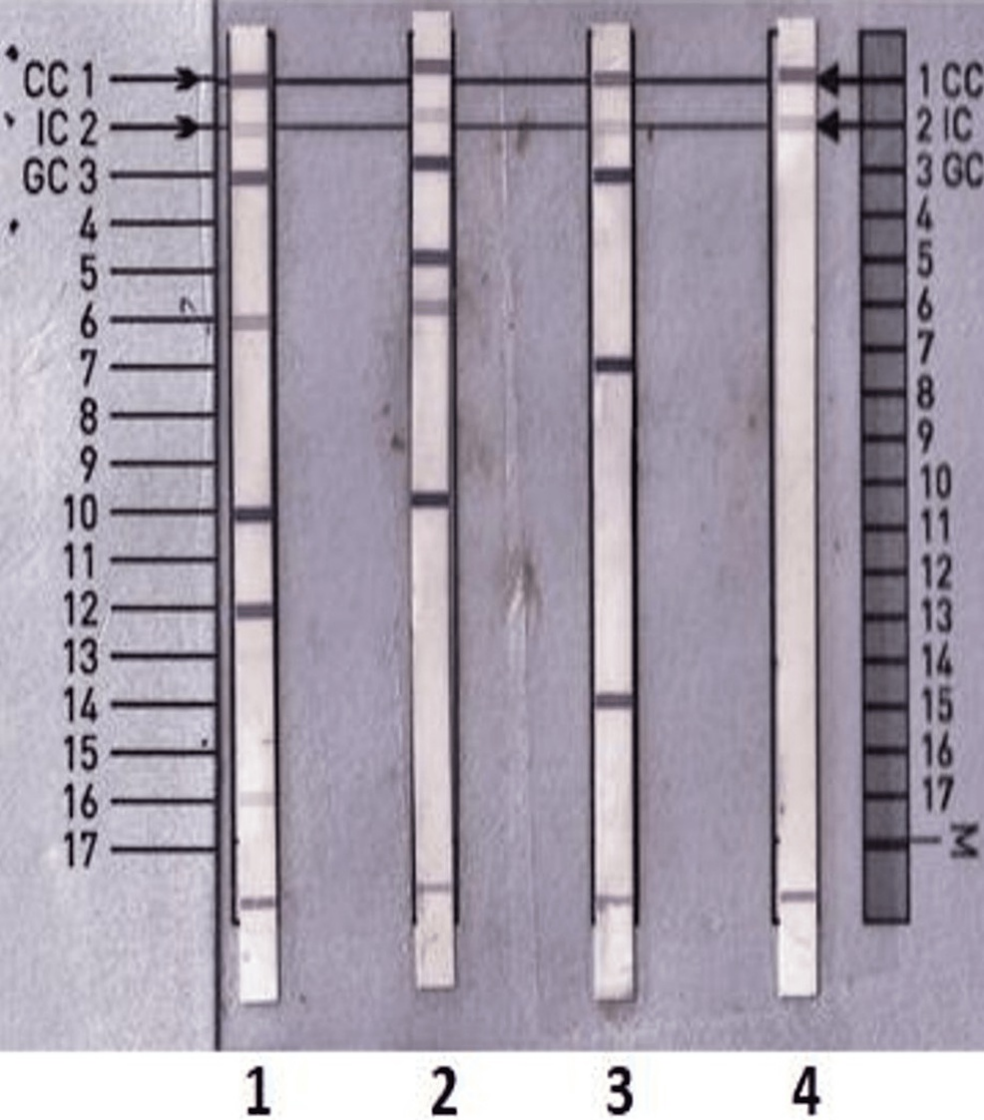
Strip of the GenoType® Mycobacterium CM assay for genotyping NTM species Isolate 1 identified as *M. kansasii* (positive control), 2 identified as *M. abscessus*, 3 identified as *M. fortuitum,* and 4 was the negative control NTM: non-tuberculous mycobacteria

In LPA, all isolates, including the negative and positive controls, a positive conjugate control line, and the universal control line, were visible, indicating the reliability of the results. As presented in Table 3, out of the 12 isolates, five (41.67%) represented patterns of *M. fortuitum*, three (25%) represented patterns of *M. abscessus* and four (33.33%) showed the pattern of *M. tuberculosis*. The positive band numbers on the CM strip for each isolate were recorded, and the patterns were evaluated to determine the species of Mycobacterium following the manufacturer's interpretation chart, as depicted in Figure 2.

**Table 3:** Distribution of NTM species among samples by the GenoType® Mycobacterium CM assay (n=08) NTM: non-tuberculous mycobacteria

NTM species (8)	Sputum	BAL	Urine	Total
*M. fortuitum* 05 (62.50%)	04 (50%)	-	01(12.5%)	05 (62.50%)
*M. abscessus* 03 (37.50%)	02 (25%)	01 (12.5%)	-	03 (37.50%)

*M. fortuitum *was detected in both sputum and urine samples while *M. abscessus* was found in sputum and bronchoalveolar lavage (BAL) samples. The distribution of various clinical specimens of NTM species is presented in Table 3.

In comparison to the Genotype Mycobacterium CM assay, the sensitivity and specificity of mPCR were 100% (95% CI; 63.06% to 100.00%) and 100.00% (95% CI; 39.76% to 100.00%), respectively. The mPCR and Genotype Mycobacterium CM assay had 100 % concordance.

## Discussion

NTM infection is a growing global health issue, exacerbated by widespread exposure to the organisms, a lack of understanding of immune susceptibility to disease, an increasing number of immune-compromised patients, time-consuming diagnostic tests, and costly, multi-drug treatment regimens that frequently fail to cure. NTM disease is typically gradual and progressive, affecting patients who are already vulnerable. There have been several epidemiological and descriptive studies of patients. However, there are still gaps in knowledge [3]. NTMs are a diverse group of mycobacteria that are ubiquitous in the environment and can cause a range of clinical manifestations. The diagnosis of TB can be challenging due to the similarities in clinical presentation and radiological findings between TB and NTM pulmonary disease. This has led to the need for more specific and sensitive diagnostic tools to identify NTM in suspected TB cases accurately. Smear microscopy cannot distinguish MTBC from NTM infection, resulting in diagnostic and clinical difficulties. The care of patients with MTBC and NTM is entirely different; thus, quick isolation, detection, and differentiation are required to manage NTM appropriately.

The prevalence of NTM in isolates from mycobacterial culture-positive patients with pulmonary TB is largely unknown [21]. However, recent studies have reported an increase in the isolation of NTM among TB suspects in different regions of the world [22,23].

The present study focuses on the molecular identification of NTM in suspected TB cases at a central India tertiary care hospital. The study used a conventional GenoType® Mycobacterium CM assay to identify species of mycobacteria in specimens from patients suspected of having TB. In this study, AFB was seen by smear microscopy in 33/400 (8.25%) isolates while 59/400 (14.75%) isolates were culture positive. However, the conventional methods are less sensitive, time-consuming, and cannot differentiate between MTBC and NTM [24]. Out of 12 SD Bioline Ag MPT64 tests and mPCR positive isolates, 08/400 (2%) NTM strains were correctly identified to species level using the GenoType® Mycobacterium CM assay. The most frequently isolated NTM species were *M. fortuitum,* followed by *M. abscessus. M. fortuitum* was found in the pulmonary (sputum) and extrapulmonary samples (urine). *M. abscessus* was found in the pulmonary samples (sputum and bronchoalveolar lavage).

A study conducted in a tertiary care hospital in Lahore, Pakistan, reported the isolation of NTM among TB patients [4]. The study used nucleic acid amplification testing for MTBC to differentiate between TB and NTM disease. The study highlights the importance of accurately diagnosing NTM disease, often misinterpreted as TB without culture and identification [25].

Correct and timely identification of NTM is crucial for patient management and infection control [22]. A laboratory-based study in South Africa aimed to identify and speciate NTM isolated from specimens submitted to a central tuberculosis laboratory throughout KwaZulu-Natal Province. The study found a high prevalence of NTM, which varied by species and geographical location [26].

A relevant study by Varghese et al. (2013) investigated the respiratory isolates collected from TB reference laboratories in Saudi Arabia from 2009 to 2010 and reported that rapidly growing mycobacteria, *M. abscessus* (31%), and *M. fortuitum *(29%), were the most common cause of NTM lung infection [27].

The findings of Harbi et al. (2014) illustrated that *M. fortuitum* (5%) and *M. abscessus* (24%) were the second most common species that caused NTM lung disease after MAC [5]. Earlier studies from North America, Europe, Asia, and Australia show that NTM infection is becoming a prominent cause of chronic lung illnesses. Similarly, high prevalences of rapidly growing mycobacteria have been observed in various areas of the globe. In several Asian nations, such as India, Taiwan, and Korea, the prevalence of quickly developing mycobacteria has been reported to approach 30% in NTM lung infections.

In this study, *M. fortuitum *was also found in urine samples. Apart from lung infections, genitourinary infections of NTM are rarely reported. Huang et al. (2010) reviewed the medical records of all patients with genitourinary NTM infections treated at the National Taiwan University Hospital from 1996-2008 and identified 15 patients having *M. fortuitum* infection [28]. 

In our study, in comparison to the Genotype Mycobacterium CM assay, the sensitivity and specificity of mPCR were 100% (95% CI; 63.06% to 100.00%) and 100.00% (95% CI; 39.76% to 100.00%), respectively. The mPCR and Genotype Mycobacterium CM assay had 100% concordance. The limitation of mPCR is that it can detect only one NTM species,* M. avium *complex, whereas the Genotype Mycobacterium CM/AS assay may identify 27 clinically significant NTM species.

In this study, four isolates were negative in the SD Bioline Ag MPT64 test, indicating the presence of NTM. Still, these four were recognized as MTBC by both molecular methods, mPCR and Genotype Mycobacterium CM assay. According to Arora et al. (2015), the SD Bioline Ag MPT-64 test can give false negative results due to the point mutation in the MPT64 gene. So, for the final confirmation of the presence of NTM, it can be done by further molecular testing [29].

The GenoType® Mycobacterium CM assay allows for rapidly and precisely identifying a wide range of Mycobacterium species. This assay has a shorter turnaround time than traditional phenotypic approaches and other molecular methods. The limitations of this test are that it requires more labor for extraction, amplification, and hybridization, and it cannot detect all species of NTM because it only includes relevant probes for the most frequent and clinically significant NTM species. Based on molecular identification using the GenoType® Mycobacterium CM assay, our study found a 2% prevalence of NTM. Nevertheless, the NTM isolation rate in India has been observed to range between 0.5% and 8.6% [30]. The GenoType® Mycobacterium CM assay, which employs multiplex PCR and reverses hybridization, performs a consistent and accurate identification test for various Mycobacterial species. The results of this experiment are simple to understand to comprehend, unlike the expertise needed to perform DNA sequencing. The GenoType® Mycobacterium CM assay is simple for differentiating Mycobacterial species in clinical settings.

The prevalence of NTM in suspected TB cases is increasing worldwide, and accurate diagnosis is crucial for patient management and infection control. Using nucleic acid amplification testing for MTBC offers improved diagnostic accuracy, compared with smear microscopy and the SD Bioline Ag MPT64 test, and assists in differentiating between TB and NTM disease. The identification and speciation of NTM can vary by species and geographical location. The study findings highlight the importance of molecular identification in accurately diagnosing NTM in suspected TB cases. Culture-based methods have been the gold standard for diagnosing mycobacterial infections, but they have limitations in their sensitivity and specificity. Molecular identification methods offer higher sensitivity and specificity for identifying NTM species.

## Conclusions

Accurately identifying NTM in suspected TB cases has critical patient management and treatment implications. NTM pulmonary disease requires different treatment regimens than TB, and misdiagnosis can lead to inappropriate treatment and potentially harmful side effects. Additionally, NTM infections are often chronic and require prolonged treatment, which can have significant economic and social impacts on patients and their families. The molecular identification of NTM in suspected TB cases is essential for accurately diagnosing and managing patients. The study conducted at a tertiary care hospital in Central India provides valuable insights into the prevalence and species distribution of NTM in this region. It underscores the need for more specific and sensitive diagnostic tools to identify NTM species accurately.
